# Antimicrobial Resistance: How Can We Overcome the Problem?

**DOI:** 10.3390/antibiotics15010082

**Published:** 2026-01-14

**Authors:** Valerio Massimo Sora, Clementine Wallet, Gabriele Meroni, Thomas Loustau, Olivier Rohr, Alfonso Zecconi, Christian Schwartz

**Affiliations:** 1 Surgical and Dental Sciences-One Health Unit, Department of Biomedical, School of Medicine, University of Milan, Via Pascal 36, 20133 Milan, Italy; gabriele.meroni@unimi.it (G.M.); alfonso.zecconi@unimi.it (A.Z.); 2 UPR CNRS 9002 ARN, IBMC, IUT Louis Pasteur, University of Strasbourg, 67300 Schiltigheim, France; clementine.wallet@unistra.fr (C.W.); thomas.loustau@unistra.fr (T.L.); olivier.rohr@unistra.fr (O.R.); christian.schwartz@unistra.fr (C.S.)

**Keywords:** antimicrobial resistance, resistome monitoring, protection strategies, alternatives to antibiotics

## Abstract

Antimicrobials are common drugs used to treat and prevent infectious diseases in plants, animals, and humans. Since their discovery in the mid-20th century, their use has dramatically increased for the benefit of humanity, and also for animal care. However, antimicrobial resistance soon appeared, which, according to the WHO, will limit or impede their use at the horizon of 2050. Indeed, antimicrobial resistance (AMR), which is a natural phenomenon in bacteria increased dramatically over the last 3 decades mainly due to the overuse and misuse of antibiotics in humans, animals, and plants. Apart from affecting human health, drug-resistant diseases also adversely affect plant and animal health, reduce agricultural productivity, and threaten food security. AMR affects all countries, regardless of economic status, and imposes high costs on health systems and national economies. Therefore, antimicrobial resistance should be studied and analyzed under the One Health paradigm. In mind of the One Health paradigm, to reduce and overcome AMR, we must take at least 3 complementary and integrated actions: (i) monitoring the resistome; (ii) developing protective strategies against antibiotic resistance; (iii) taking curative actions by designing new and original treatments. Moreover, the three actions must be conducted simultaneously due to the continuous adaptation of bacteria.

## 1. Introduction

Antimicrobials are drugs used to treat and prevent infectious diseases in plants, animals, and humans. In medicine, antibiotics are among the most effective types of medication. However, a rising number of infections that are resistant to antimicrobial molecules is compromising the effectiveness of antibiotics. One of the biggest threats to global public health is antibiotic resistance, which is linked to higher rates of morbidity and mortality as well as higher treatment costs. The severity of the issue has prompted several national (e.g., EU member states) and international (e.g., FAO and OIE) organizations to take action to protect the public [1,2]. It is forecasted that 1.91 million people could potentially die as a direct result of antimicrobial resistance (AMR) in 2050, an increase of almost 70% per year compared to 2022 [3]. Over the same period, the number of deaths in which AMR bacteria play a role will increase by almost 75% from 4.71 million to 8.22 million per year [3]. Resistance to antimicrobials, including antibiotics, renders medications ineffective, complicating or preventing infection treatment. It elevates the risks of disease spread, severe illness, disability, and mortality. AMR is a natural phenomenon arising from genetic changes in bacteria. However, human activities—particularly the overuse and misuse of antibiotics in humans, animals, and plants—accelerate its emergence and spread [1]. Drug-resistant diseases adversely affect plant and animal health, reduce agricultural productivity, and threaten food security. AMR affects all countries, regardless of economic status, and imposes high costs on health systems and national economies. Vulnerable populations and those in low-resource settings are most severely impacted. Factors contributing to the spread of AMR include poor sanitation and hygiene in humans and animals, inadequate disease prevention and control measures, lack of awareness, and weak enforcement of relevant laws [1]. The 2022 Global Antimicrobial Resistance and Use Surveillance System (GLASS) report highlights alarming rates of resistance in common bacterial infections. Of major concern is the median rates reported across 76 nations: 35% of *Staphylococcus aureus* infection is methicillin-resistant, and 42% of infections due to *Escherichia coli* are resistant to the newest third-generation cephalosporin [4]. Consequently, it is increasingly challenging to effectively treat common infections. Managing antimicrobial resistance will need complementary and integrated actions, as seen in the WHO’s global action plan on antimicrobial resistance, which includes:

1. Description, collection, and follow-up of antibiotic resistance genes or resistome.

2. Preventive actions by developing protection strategies that consider the fact that antimicrobial resistance is an interconnected problem between human, animal, and environment, also known as a “One Health issue”.

3. Curative actions by developing alternatives to the current antibiotics, such as phage therapy, nanoparticles, antimicrobial peptides, and the use of artificial intelligence to discover new antibiotics.

## 2. What Is Antimicrobial Resistance and How Is It Acquired?

**Figure 1:**
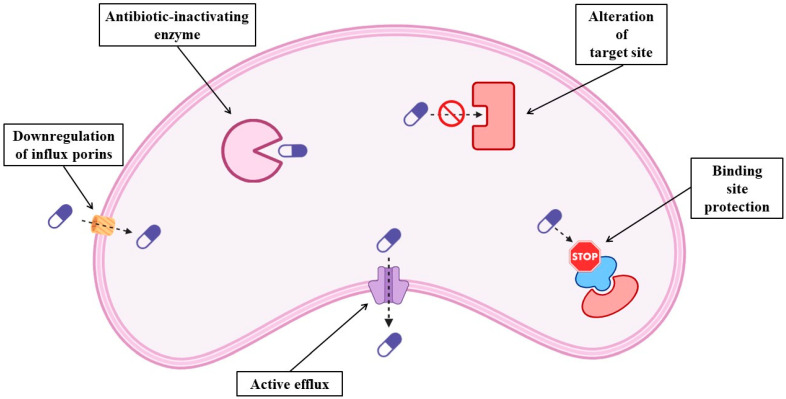
Schematic representation of the molecular mechanisms of antibiotic resistance in bacteria.

The term resistome encompasses all known and unknown antibiotic resistance genes globally. Antibiotic resistance, a persistent challenge, is escalating in severity. Besides the widespread use of antimicrobials to treat human diseases, overcrowding, increased worldwide movement, increased use of antibiotics in livestock production and clinical settings, selection pressure, poor sanitation, wildlife spread, and inadequate sewage disposal systems are the causes of the emergence of the global resistome [5,6]. Antibiotic resistance is a One Health issue that needs monitoring and control in human medicine, animal husbandry, agriculture, and aquaculture [7]. Numerous organizations and institutions worldwide have acknowledged that AMR is a major public health issue. Though many studies treat the problem and offer numerous resolutions, not much progress has been made to date. Sadly, the rise in antibiotic resistance is a recurring problem [8]. It is important to consider that the emergence of antibiotic resistance is a natural adaptive response and an obvious example of Darwin’s theories of evolution. One of the most important developments in modern medicine has been the introduction of antimicrobial therapy into clinical practice. Several advanced and intricate medical procedures have been invented that have greatly increased life expectancy in the world. On the other hand, bacteria have evolved sophisticated and inventive ways to elude the antibiotic onslaught, which were probably accelerated using antimicrobials in clinical practice. Antibiotic resistance has rapidly evolved in the past few decades to become one of the greatest public health threats of the 21st century [9]. Bacterial resistance can be classified as innate resistance or acquired resistance. Innate resistance is the capacity of a bacterium to withstand the effects of a particular antibiotic because of its innate structural or functional characteristics, while acquired resistance may result from the acquisition of new genes by horizontal gene transfer [9]. AMR is primarily caused by alterations within the bacteria (innate resistance) [10], and can happen in several ways, e.g., overexpression of enzymes inactivating the antibiotics, mutations in the target site protecting the target from the effects of the antibiotics, downregulation of porins affecting influx, and active efflux of antibiotics (Figure 1). These changes may arise from point mutations or chromosomal changes in regulatory genes or chromosomal structural elements, giving rise to new resistant strains [11].

In acquired resistance, plasmids and other Mobile Genetic Elements (MGEs) are implicated in many cases [12]. These MGEs are transmitted between different bacterial species by horizontal gene transfer (HGT) through different mechanisms described elsewhere [13,14]. Selective pressure on the environment permits the survival and multiplication of organisms with unique mutations or newly evolved features [15]. Antibiotic stewardship, defined by WHO as “a systematic approach to educate and support health care professionals to follow evidence-based guidelines for prescribing and administering antimicrobials” [16], plays an important role in the development of AMR. Physicians, when prescribing a wide-spectrum or “just in case” antibiotic when a specific narrow-spectrum antibiotic might be a better fit because of incomplete or erroneous information, accelerate the development of antibiotic resistance [17,18]. Inappropriate use of antibiotics in viral infections, misuse by patients, or antibiotic self-medication also contribute to the buildup of antimicrobial resistance in bacteria [10,19]. In hospitals, if policies and procedures in place are not adequate to keep the healthcare facilities clean, AMR’s genesis and spread are facilitated [20]. Both industrialized and developing countries use antibiotics as growth supplements and growth boosters for their animals, even if this practice has been forbidden in many countries. Similarly, antibiotic-treated animals will develop antibiotic-resistant microorganisms. Food chains can easily spread antibiotic-resistant bacteria [21,22]. Animal manure also contributes to the spread of antibiotic-resistant bacteria across the ecosystem [23,24,25]. Given its complex transmission network, antimicrobial resistance should be studied and analyzed under the One Health paradigm [1]. According to the One Health paradigm, at least three complementary and integrated actions are needed to reduce the threat of AMR: (i) monitoring the resistome (ii) prevention of antibiotic resistance (iii) curative actions by designing new and original treatments. The three actions must be conducted simultaneously due to the continuous adaptation of bacteria.

## 3. Environmental Resistome and Its Monitoring

The environmental resistome (ER) comprises antibiotic resistance genes of bacteria found in soil, water, and air. ER epidemiology gives insight into how environmental resistance genes arise, proliferate, and affect public health by interacting with the microbiomes of humans and animals. Human activities, including extensive antibiotic use in healthcare, aquaculture, agriculture, or wastewater treatment facilities (WWTPs), where antibiotics are frequently used, impact the ER [26,27,28,29]. Resistance is spreading through environmental pollution and food chains affecting wildlife. According to some studies, the abundance of AMR genes in soil samples has increased by 2 to 15 times since the 1970s, particularly genes that encode for tetracycline and β-lactam resistance [28]. Untreated wastewater and animal manure are important sources of AMR genes in low-to-middle-income countries (LMICs), where environmental contamination is made worse by inadequate sanitation and overuse of antibiotics in agriculture. Aquaculture and high-density animal husbandry have a major role in the spread of AMR. It is known that residual antibiotics found in aquaculture effluents enable human antibiotic-resistant infections [28,30]. Aquatic habitats are contaminated by antibiotics and resistant bacteria from untreated wastewater. Even at modest concentrations, environmental antibiotics can exert selection pressure that preserves and disseminates resistance genes. ER is not limited to aquatic systems. Dust particles and wastewater aerosols containing microbes in both urban and rural environments also play a role in the spread of AMR genes [31]. Public health is seriously threatened by the rise in environmentally resistant organisms. Food, water contaminated by resistant bacteria, or direct contact with the polluted environment can infect human populations. For example, swimmers exposed to resistant *Escherichia coli* in polluted waters have been shown to enhance the risk of transmitting AMR genes [30]. Furthermore, farmworkers and consumers are also exposed to AMR bacteria found in the manure of antibiotic-treated animals used in agricultural soils. Likewise, controlling the spread of resistant bacteria becomes more difficult as AMR genes might go up the food chain to consumers from crops that are irrigated with polluted water [28]. A One Health strategy that incorporates environmental, animal, and human health is necessary for the effective management of the environmental resistome [32]. Metagenomics-based surveillance systems or direct actions are used to monitor AMR genes in various environmental reservoirs. Several laboratories, including ours, have used molecular approaches, including PCR, serotype identification, and whole-genome sequencing to analyze the genomic features and transmission of AMR genes of pathogens [33,34]. We notably showed that Shiga-Toxin Producing *E. coli* (STEC) isolates were resistant to many antibiotics, especially to ampicillin [33]. The study provides early warning of the prevalence and spread of AMR genes. Other epidemiological approaches focused on other common pathogens, including streptococci and staphylococci [32,35], the latter being listed by the Center for Disease Control (CDC) as one of the most virulent and resistant to antimicrobial agents [36,37]. These studies established a link between isolates from animal and human populations and highlighted the importance of using a global approach to learn more about the spread of pathogens and their AMR genes. Mitigating the spread of AMR genes requires reducing the use of antibiotics, efficient wastewater treatment, and boosting the biosecurity of aquaculture and agriculture. Targeted measures like better water treatment and stronger laws on the use of antibiotics in agriculture are crucial, especially in low- and middle-income countries where waste management and sanitation infrastructure are frequently lacking. Because antibiotic resistance is a worldwide issue, establishing comprehensive policies must consider the migration of AMR genes between areas, which is aided by human mobility, commerce, and environmental degradation [30,31].

## 4. Strategies to Prevent Anti-Microbial Resistance

Antimicrobial resistance requires multifaceted protection strategies addressing the issue from several angles, including public awareness, agricultural use, and medical procedures. Healthcare practitioners should prioritize appropriate diagnostic and prescribing practices to decrease the unnecessary use of antibiotics. Narrow-spectrum antibiotics, when inappropriately prescribed, foster resistance. Enforcing restrictions on narrow-spectrum antibiotics may reduce the selective pressure on resistant bacteria. The use of broad-spectrum antibiotics is not a solution either, since in this case resistant strains may emerge more likely [17]. Stringent hygiene measures, such as hand washing and wearing masks, are crucial in hospitals to stop the spread of resistant strains. Surveillance systems that monitor infection rates are also essential [20]. Public health campaigns educating the public about the risks of self-medication and the importance of completing prescribed antibiotic regimens may increase community participation in AMR prevention efforts [38]. The agriculture sector must minimize the use of antibiotics as growth promoters for animals. Laws encouraging alternatives such as improved animal husbandry, biosecurity measures, and vaccination programs can lower the need for antibiotics while maintaining animal health and productivity [5]. Environmental management is even more crucial as efficient waste treatment methods may help reduce the quantity of antibiotics and thus the emergence of resistant microorganisms in the ecosystems. This includes appropriately disposing of pharmaceutical waste and adhering to legislation preventing antibiotic waste disposal in agricultural regions [28]. According to Ding et al. [31], promoting sustainable aquaculture practices may reduce the use of antibiotics in fish farming, a primary contributor to the ER. Usually, antibiotics are directly released into the water, creating the perfect condition for sub-optimal concentration exposure and consequent resistance buildup to the specific molecule. Overall, encouraging a One Health approach that integrates perspectives on human, animal, and environmental health is crucial to developing comprehensive strategies to combat AMR globally. This approach recognizes AMR as a complex issue requiring collaboration from the public, environmental scientists, veterinarians, medical professionals, and lawmakers [8]. Creating interdisciplinary task teams focused on AMR monitoring, research, and education across all sectors can help to establish new targeted treatments. Phage therapy, the use of new antimicrobial peptides, and vaccinations may provide new alternative therapeutics [9].

## 5. Alternative Treatments to Fight Anti-Microbial Resistance

It is imperative to develop alternative strategies to antibiotic therapies that are safe and more effective against infectious pathogens. In this review we consider alternative strategies such as phage therapy, the use of nanoparticles (NPs), effective antimicrobial peptides (AMPs), and artificial intelligence (AI) to develop new antimicrobial molecules.

### 5.1. Phage Therapy

Today there is a huge interest in substituting antibiotic treatment with phage therapy. Despite several obstacles, bacteriophage therapy has the potential to replace antibiotics in the future for treating drug-resistant infections. Phage therapy was invented in Georgia in the early 1920s and later reached Eastern and Western Europe [39]. Bacteriophages have several characteristics that set them apart from other antibacterial agents. They encode lysins or other antimicrobial peptides that specifically destroy bacterial species. Because of their capacity to infect susceptible bacteria, living phages can naturally transport modified genes encoding antimicrobial compounds. For systemic infections, the treatment can be administered intravenously or topically to open wounds [40,41]. Nevertheless, phage treatment has significant limitations. Their high selectivity for host bacterial species is a primary limitation prohibiting their use as an empirical treatment for acute infections. Another major limitation is the inability of the phage to bind and infect a broad range of clinical strains. Clinical strains may acquire resistance to phage by different mechanisms, e.g., mutations of receptors involved in the uptake of phage or through the CRISPR-Cas systems [42,43]. To overcome these limitations, recent advances in genetic engineering have greatly improved the use of phage therapy. For example, genome engineering can be used to redirect phage specificity by modifying the structure of domains of the phage protein involved in the binding of bacterial receptors [44]. The study of phage-host interactions has benefited from recent advances in the use of artificial intelligence (AI). Importantly, it has accelerated the screening of a panel of phages for each clinical isolate [45]. Indeed, several groups have made promising use of AI to predict strain-level interactions between receptor-binding phage proteins and bacterial receptors [46,47]. Another way to overcome the use of phage is to use purified lysins. These highly selective peptidoglycan hydrolases, known as enzybiotics, can lead to rapid osmotic lysis and bacterial death by degrading the bacterial cell wall [48]. The employment of bioengineered lysins with intended features, such as increased lytic activity or broader spectrum bacteriophage lysins, is promising due to their modular nature. For example, genetically modified lysins can be used to eliminate Gram-negative bacterial infection. The enzymes do not cause an immunological reaction and are unlikely to cause resistance. These appealing qualities make enzybiotics effective and accessible agents to combat antimicrobial resistance [49]. Another lysin, the murein hydrolase from the streptococcal bacteriophage C1, exhibited great specificity and efficacy while not harming other naturally occurring microbes, as it rapidly eliminated group A streptococci both in vitro and in vivo [50]. Briers et al. showed that the fusion of lysins to cationic AMPs permeabilized the outer membrane of *P. aeruginosa* and induced the breakdown of the peptidoglycan layer both in vitro and in vivo [51]. The use of bacteriophage therapy is on the rise, mostly because of the AMR problem. Recommendations for regulatory conditions for the therapeutic use of phages include strictly lytic phages, demonstrated antibacterial activity against the target pathogen and removal of bacterial debris and endotoxins [52].

### 5.2. Metal Nanoparticles in Biomedical Science

Metal nanoparticles (NPs) make up a broad class of materials that contain compounds with at least one dimension less than 100 nm. These materials can be 0D, 1D, 2D, or 3D, depending on their general geometry [53]. In this review, we will focus on metal NPs. Metal NPs are composed of three layers: (a) the surface layer, functionalized with substances such as surfactants, metal ions, small molecules, and polymers; (b) the shell layer, chemically distinct from the core; and (c) the core, the main component of the NP [54]. Although the mechanism of antibacterial action of NPs is not well understood, it is suggested that five major characteristics of the NPs influence the interaction with bacteria either positively or negatively: (i) shape, (ii) size, (iii) surface-volume ratio, (iv) concentration, and (v) electrostatic forces that drive the attraction between bacteria and nanoparticles. Indeed, when NPs increase in size, both their stability and the surface-to-volume ratio, factors that govern interaction with the cell wall and therefore affect antimicrobial efficacy, diminish [55]. Most bacteria possess a negatively charged cell wall, which facilitates the attraction and physical binding of positively charged molecules. Following the initial interaction, metal nanoparticles can compromise the cell wall by attaching to and modifying proteins and lipids (thereby affecting osmotic equilibrium), infiltrate the microorganism, and damage internal structures by binding to negatively charged proteins and nucleic acids [56]. Antimicrobial compounds can be delivered by nanoparticulate materials, or they may already contain antimicrobial substances. The antibacterial, antiviral, and anticancer properties of metal and metal oxide-based nanoparticles and antibiotics, along with their lower toxicity, make them attractive therapeutic options for use in the biomedical sciences. Apart from serving as vehicles for specific drug delivery, nanoparticles can also exhibit other antibacterial behaviors, including bacterial wall disruption, biofilm inhibition, host immune response modulation, reactive oxygen species generation, and damage to essential DNA and protein molecules of resistant bacteria. Nanoantibiotics could probably be useful against bacteria that are resistant to antibiotics because of their various mode of action. However, to guarantee the effectiveness and safety of the nanoantibiotics in clinical settings, thorough investigation of their pharmacokinetics and specificity must be carried out [57]. We might also use NPs to treat wastewater in controlled conditions to attenuate the spread of antimicrobial resistance. Indeed, it was shown that waste waters containing NPs eliminated 45.0% to 62.0% of antibiotic-resistant bacteria [58]. This was primarily due to the damage of cell membranes by cell membrane-bound NPs and by inducing reactive oxygen species (ROS) killing bacteria [58]. The potential use of metallic nanoparticles for the removal of antibiotic-resistant bacteria and antibiotic resistance genes from wastewater is discussed in recent publications [59,60]. In addition, silver nanoparticles (AgNPs) have emerged as a promising alternative to traditional antimicrobial agents [61]. Zhao and colleagues’ recent investigation on the co-administration of NPs and antibiotics showed that in bacteria, treated with cyclic ciprofloxacin and silver ions (Ag+) alternately, resistance did not appear [62]. Another recent study strongly corroborates this result, confirming that consecutive AgNP applications render bacteria more susceptible to 38 antibiotics [63]. These results open new avenues in using antibiotics even for pathogenic bacteria that have already developed resistance. Finally, a study by Roy et al. showed that the antibacterial activity of aminoglycosides, β-lactams, glycopeptides, and cephalosporins against Methicillin-resistant *Staphylococcus aureus* considerably increased in the presence of sublethal quantities of TiO_2_ NPs [64].

### 5.3. Antimicrobial Peptides

It was shown that certain small naturally occurring peptides (AMPs) exhibit potent antimicrobial activities against various pathogens [65,66]. These antimicrobial peptides (AMPs) play a crucial role in the innate immune system of many organisms and are being explored as potential alternatives to traditional antibiotics [67,68,69]. Among AMPs’ various targets are intracellular and plasma membrane proteins of pathogenic bacteria, making AMPs a promising substitute for antibiotics [68,70,71,72,73]. Cationic AMPs work better since some anionic components, such as lipoteichoic acid in Gram-positive bacteria or lipopolysaccharide (LPS) in Gram-negative bacteria, are present in the plasma membranes of bacteria. AMPs can permeate the membrane to perform intracellular functions or perforate it to cause intracellular contents to seep out. AMPs’ non-specific effects on bacteria reduce the emergence of resistance. A broad class of cationic small peptides (AMPs) between 30 and 60 amino acids that are extracted from bacteria are called bacteriocins. They are divided into two groups: peptides produced by non-ribosomes with broad antibacterial activity and peptides synthesized by ribosomes with relatively narrow antibacterial activity against bacteria and fungi [74]. In the *Streptomyces albofaciens* culture broth, Wang et al. identified a novel short non-ribosomal AMP called albopeptide 6, which exhibited narrow-spectrum action against vancomycin-resistant Enterococcus faecium [75]. Nisin, a distinct member of the bacteriocins family, was shown to have potent antibacterial activity against a range of Gram-positive and even Gram-negative bacteria [76]. Tong et al. found that the combination of nisin and penicillin or chloramphenicol was potent against *E. faecalis*, whereas the use of either antibiotic alone had no discernible effect [77]. Combining antibiotics and AMPs can be a therapeutic strategy to combat antibiotic resistance, increase the effectiveness of medicines, and reduce the toxicity or side effects of high-concentration antibiotics. The combined treatment can impair intracellular ion homeostasis, enhance bacterial membrane permeability, and reduce antibiotic efflux, all of which prevent the formation of biofilms and bacterial survival [78]. For example, Li et al. showed that the antimicrobial peptide SAAP-148 and the tetracycline antibiotic demeclocycline hydrochloride (DMCT) work synergistically against the multidrug-resistant (MDR) *P. aeruginosa* strains PAO1 and ATCC27853 [79]. It is also possible to obtain synergistic antimicrobial effects by mixing AMPs with other substances or medications. Nisin and citric acid together had superior antibacterial action against *S. aureus* and *L. monocytogenes* because they increased cell damage and component release [80]. As a result, either used alone or in combination, AMPs provide a novel alternative to treat bacteria that are resistant to antibiotics. We assume that AMPs with broad-spectrum antibacterial activities may replace conventional antibiotics in the future. The Food and Drug Administration (FDA) has currently approved three AMPs for bacterial infection, and three more AMPs are undergoing clinical research [81]. Moreover, as described in the following section, the use of AI will help us to identify and design new peptides with optimal therapeutic properties [82].

### 5.4. Artificial Intelligence-Assisted Generation of New Antimicrobial Molecules

Developing new antimicrobial molecules is a labor-intensive, costly, and failure-prone process, often taking up to ten years and hundreds of millions of dollars [83]. Between 2014 and 2019, only 14 new antibiotics were developed and approved [84]. Finding novel medications with new mechanisms of action will require computer-aided prospection to expedite the identification of antibiotics [85]. 1030–1060 drug-like compounds are thought to exist [86], even though there are 20n variations for every n-length canonical amino acid sequence. Although there are many opportunities for computational antibiotic design in this vast combinatorial space, a thorough search is not feasible within a reasonable timeframe. These difficulties highly motivate the creation of effective algorithms for high-throughput antibiotic discovery. Any AI-driven project starts with collecting the experimental data needed to create a model. Next, the data are translated into representations that are ready for AI. After that, models are trained with algorithms that might be anything from cutting-edge neural networks to conventional decision trees. Lastly, trained models can be used to forecast a variety of characteristics, such as an antibiotic’s efficacy, its propensity for hazardous action, the emergence of resistance, or the structure of new compounds with desired properties [87]. Machine Learning (ML) models can determine the pathways involved in cytotoxicity, the mechanisms of resistance, and the mechanisms of action of antimicrobial drugs with unknown roles [88]. An outstanding work from Yoshida et al. demonstrated how a combined use of genetic algorithm, machine learning, and in vitro evaluation can improve the antimicrobial activity of already known peptides against *Escherichia coli*. Briefly, two libraries of peptides have been generated with extremely high identity to the wild-type peptide. Following synthesis, all the peptides of the two libraries were tested for their antimicrobial activities against *E. coli*. A fitness matrix was created comparing the antimicrobial activities with the different mutations present in the peptides. A new library of peptides was generated starting from the highest-ranking peptides of the previous library, and then the process of synthesis and antimicrobial activity testing was reiterated. At the end this process produced a total of 45 peptides with a 20-fold higher activity than the wild-type peptide, showing the efficacy of this method in finding good candidates for new antimicrobial peptides [89]. The work of Stokes and colleagues [90], who identified a potential candidate chemical for broad-spectrum antibacterial action using a composite in silico and empirical technique, offers another proof of the use of AI in the discovery of new antimicrobial compounds. In short, scientists used a set of 2335 molecules to train a deep neural network model that predicted the inhibition of *E. coli* growth. The resulting model was then applied to several chemical libraries to find possible lead compounds that exhibited activity against. Following the ranking of the compounds based on the predicted score of the model, a list of candidates was chosen based on availability, chemical structure, and a pre-specified prediction score threshold. Following these in silico procedures, they discovered that the chemical known as halicin—structurally distinct from traditional antibiotics—is a strong inhibitor of *E. coli* growth. Additional empirical testing showed that halicin selectively dissipates the ΔpH component of the proton motive force, exhibiting growth-inhibitory characteristics against a broad phylogenetic spectrum of pathogens. This action was further validated in an in vivo murine model. This study demonstrated the substantial influence of machine learning on the discovery of antibiotics by reducing screening costs and improving lead compound identification accuracy at the same time [90]. A summary of the main alternative strategies to fight antimicrobial resistance treated in this review can be found in the table below (Table 1).

## 6. Conclusions

AMR is becoming a global health crisis that requires fast and comprehensive action. The abuse and misuse of antibiotics in human medicine, agriculture, and animal husbandry exacerbates antibiotic resistance, which is not simply caused by spontaneous bacterial evolution, according to the literature. This occurrence shows how Darwinian evolution can cause bacteria to develop resistance mechanisms, which challenge antibiotics. Innate and acquired bacterial resistance mechanisms, such as enzymatic antibiotic degradation, target site change, and efflux pump activation are normal adaptive responses but are accelerated by humans. Facing this important threat we believe 3 main actions, following what has already been stated by the WHO [1], are crucially needed and complementary:

1. Epidemiological study of the environmental resistome. This approach is needed to establish policies to reduce the misuse of antibiotics or to eliminate antibiotics from wastewaters. Molecular biology tools, e.g., sequencing, have been used to survey viral emergence in wastewater [91,92]. We suggest a similar approach to surveying antibiotic resistance in wastewater.
2. Preventive actions by developing protection strategies to combat antibiotic resistance (stringent hygiene measures in hospitals, enforcing restrictions for narrow-spectrum antibiotics, etc.)
3. Curative actions by developing alternatives to current antibiotics. In Table 1, we summarize the advantages and inconveniences of these alternative strategies to fight antimicrobial resistance.

Our paper outlines the main challenges of AMR, but it also summarizes emerging options that may reduce the global threat. Appropriate use of AI in pharmaceutical discovery and alternative therapies offers hope for addressing antibacterial therapy disadvantages. These measures must be supplemented with worldwide efforts to tighten regulatory frameworks, create awareness, and promote management to stop antibiotic resistance.

## Figures and Tables

**Table 1:** Advantages and disadvantages of the alternative strategies to fight antimicrobial resistance analyzed in this review.

Alternative Strategy	Advantages	Disadvantages	References
Phage therapy	High specificity. Topical and intravenous treatment.	Narrow spectrum of action. Prohibited use for empirical treatment.	[36,37,38,39,40,41,42,43,44,45,46,47,48,49]
Nanoparticles	Broad-spectrum. Can be combined with antibiotics to enhance efficacy. Low probability of resistance build-up.	Long-term toxicity unknown. High costs of production.	[50,51,52,53,54,55,56,57,58,59,60,61]
Antimicrobial peptides	Broad-spectrum. Synthetic enhancement of stability and efficacy. Low probability of resistance build-up.	Susceptible to in vivo degradation. High costs of production. High concentrations may exert toxicity.	[62,63,64,65,66,67,68,69,70,71,72,73,74,75,76,77,78,79]
Artificial intelligence	Quick drug discovery. Resistance patterns prediction. Efficacy enhancement of already existing treatments.	Requires high computing power. Highly dependent on dataset quality and completeness. Large amount of data required to train models.	[80,81,82,83,84,85,86,87]

## Data Availability

No new data were created or analyzed in this study. Data sharing is not applicable to this article.

## Abbreviations

The following abbreviations are used in this manuscript:

AMR	Antimicrobial resistance
GLASS	Global Antimicrobial Resistance and Use Surveillance System
HGT	Horizontal gene transfer
MGE	Mobile genetic element
ARG	Antibiotic resistance gene
WWTP	Wastewater treatment plant
LMIC	Low-to-middle-income country
PPE	Personal protective equipment
NP	Nanoparticle
AMP	Antimicrobial peptide
AI	Artificial intelligence
ROS	Reactive oxygen species
Ag^+^	Silver ions
TiO_2_	Titanium dioxide
LPS	Lipopolysaccharide
DMCT	Demeclocycline hydrochloride
MDR	Multidrug-resistant
FDA	Food and drug administration
MOA	Mechanism of action
CADD	Computer aided drug design
ML	Machine learning
